# Using formative evaluation of a community-based opioid overdose prevention program to inform strategic communication for adoption, implementation, and sustainability

**DOI:** 10.1186/s12889-023-15229-2

**Published:** 2023-02-15

**Authors:** Candice Bangham, Stacey Cunnington, Sarah Fielman, Meredith Hurley, Sarko Gergerian, Jacey A. Greece

**Affiliations:** 1 Department of Community Health Sciences, Boston University School of Public Health, 801 Massachusetts Avenue, 02118 Boston, MA USA; 2 Winthrop Department of Public Health & Clinical Services, 45 Pauline Street, 02152 Winthrop, MA USA; 3 Winthrop Police Department, 3 Metcalf Square, 02151 Winthrop, MA USA

**Keywords:** Consolidated Framework for Implementation Research, Public health programs, Public safety, Opioid programs, Intervention, Diffusion of innovation, Implementation frameworks, Formative evaluation, Multi-sector partnerships

## Abstract

**Background:**

Opioid addiction and overdose is a public health problem in the United States and is expected to remain with substance use increasing due to the COVID-19 pandemic. Communities that approach this issue through multi-sector partnerships experience more positive health outcomes. Understanding motivation for stakeholder engagement in these efforts is essential to successful adoption, implementation, and sustainability particularly in the shifting landscape of needs and resources.

**Methods:**

A formative evaluation was conducted on the C.L.E.A.R. Program in Massachusetts, a state heavily impacted by the opioid epidemic. A stakeholder power analysis identified appropriate stakeholders for the study (*n* = 9). The Consolidated Framework for Implementation Research (CFIR) guided data collection and analysis. Surveys (*n* = 8) examined perception and attitudes on the program; motivations and communication for engagement; and, benefits and barriers to collaboration. Stakeholder interviews (*n* = 6) explored the quantitative findings in more detail. Surveys were analyzed using descriptive statistics and a content analysis with deductive approach was conducted for stakeholder interviews. The Diffusion of Innovation (DOI) Theory guided recommendations for communications to engage stakeholders.

**Results:**

Agencies represented a range of sectors and the majority (*n* = 5) were familiar with the C.L.E.A.R. Program. Despite the many strengths of the program and existing collaboration, based on the coding densities of each CFIR construct stakeholders identified crucial gaps in the services the program provided and noted that the overall infrastructure of the program could be enhanced. Opportunities for strategic communication to address the stages of DOI align with the gaps identified in the CFIR domains to result in increased agency collaboration and expansion of services into the surrounding communities to ensure sustainability of the C.L.E.A.R. Program.

**Conclusions:**

This study explored factors necessary for ongoing multi-sector collaboration and sustainability of an existing community-based program especially given the changing context from COVID-19. Findings informed both program revisions and communication strategies to promote the program to new and existing collaborating agencies and the community served, and identify effective communication approaches across sectors. This is essential for successful implementation and sustainability of the program, especially as it is adapted and expanded to address post-pandemic times.

**Trial registration:**

This study does not report results of a health care intervention on human participants, however it was reviewed and determined an exempt study with the Boston University Institutional Review Board (IRB #H-42107).

**Supplementary Information:**

The online version contains supplementary material available at 10.1186/s12889-023-15229-2.

## Background

Opioid addiction and overdose is a pervasive public health problem in the United States [1], and Massachusetts is heavily affected [2]. There are many behavioral and environmental determinants including past or current substance use, untreated psychiatric disorders, younger age, and social or family environments that encourage misuse [3]. Certain communities are affected more than others [4], necessitating multi-sector, community-based approaches [5–7] to ensure positive outcomes [8]. Communities disproportionately impacted by opioid addiction and overdose include veterans [9], specific racial/ethnic groups [10], incarcerated individuals [11], urban-located [12], and those experiencing homelessness or who are vulnerably housed [13]. The combined efforts of public health agencies, public safety agencies, law enforcement, mental health services, and various recovery resources are integral for successful adoption and implementation of comprehensive, multi-sector, community-based opioid overdose interventions [1, 6, 7, 14–16] to ensure positive outcomes through increased collaboration, awareness, and resource allocation.

Recognizing this, in 2010 a national drug policy was developed in the United States that sought to create a “balanced public health and public safety approach”. At the core of this policy was the recognition of addiction as a disease which should be treated in lieu of punishment [17]. Accordingly, as part of the National Drug Control Strategy there were specific initiatives in communities that aligned public health and safety efforts to foster collaboration between public safety and public health to prevent drug use as well as to expand community-based recovery support programs such as peer-led programs, recovery schools, and mutual help groups to increase access to resources [18]. As a result of this policy, an exemplar model using multi-sector collaboration was developed to strengthen community-based recovery support programs [19]. This promising model employs police-referral programs that utilize trained peer recovery coaches (PRC) and police personnel to conduct outreach following an overdose. Research has demonstrated the positive effects of peer recovery support in decreasing risky behaviors and increasing treatment utilization [20] with police-referral programs receiving federal support [21] and attention for national partnerships [22] as they divert individuals away from the justice system and into effective treatment models. In addition, preliminary research suggests that PRC utilization significantly decreases substance use and suggests those who utilize PRC services increase connections to health care and behavioral health services [23].

Presently, the COVID-19 pandemic has not only propelled the importance of addressing opioid overdose but highlighted the need to engage multiple sectors for success. Rates of overdose are growing with an increased reliance on substances due to the interruptions and stressors to daily life [24]. Community-wide efforts to reduce opioid overdose will be even more important in the coming years as communities rebuild with multi-agency collaboration including prevention, medical care, education, and mental health care [5, 6, 16] necessary. For example, one recent effort is the use of test strips, which is a demonstrated harm reduction intervention measure to reduce the risk of a fentanyl-induced overdose [25]. Additionally, naloxone has been shown to be critical in reversing opioid overdoses [26] as well as naloxone distribution has strong efficacy for reducing fatal overdoses [27]. Understanding community needs, identifying resources to address the needs [28], translating findings into targeted programming, and communicating opportunities for engagement to stakeholders ensures diffusion of efforts and supports populations most at risk [28, 29].

The Community and Law Enforcement Assisted Recovery (C.L.E.A.R.) Program, established in 2014 and not since formally evaluated, is a community-based program local to Winthrop, MA that aligns public health and safety to assist those who have experienced opioid overdose with accessing recovery resources. Aligned with principles outlined in the National Drug Control Strategy [19] the C.L.E.A.R. Program aims to address opioid overdose through multi-sector partnerships that result in the following goals: 1) shared understanding of the problem and needs in the community, 2) optimized capacity of a collaborative response to address opioid overdose, and 3) shared accountability of the efforts and resources to continually respond to the shifting landscape of substance use. The C.L.E.A.R. Program team consists of a licensed mental health clinician outreach officer, a certified peer recovery coach [19, 20], a peer recovery coach supervisor, and a licensed clinical social worker [30]. The team administers a “door knock” at a residence following an overdose to offer support and resources and relies on the police-referral model [19] to lead individuals to treatment. The program has adapted in recent years with the pandemic. For example, in response to the unavailability of primary mental health care during the COVID-19 pandemic, the C.L.E.A.R. Program team sent naloxone and fentanyl test strips to those identified as at risk for an overdose or those who have overdosed in the past. These adaptations and enhancements continue as the needs of the community evolves and as the context and resources change, warranting ongoing evaluation particularly through formative evaluation, which is essential to identify factors related to program implementation that lead to meaningful findings and recommendations [31].

Public health and public safety partnerships have been highlighted for almost two decades and heightened social awareness coupled with COVID-19 impacts have elevated critical need for multi-sector collaboration in communities [32, 33]. With proper tools, resources, and training, and multi-sector collaboration may effectively address substance use issues [34]. While the C.L.E.A.R. Program was established prior to the pandemic, there is a need to adapt the program to the COVID-19 context, which offers an opportunity to examine gaps in services and revise the program for successful adoption, ongoing implementation, expansion, and sustainability [28, 29, 35].

Formative evaluations allow planners to uncover strengths and limitations of the program and provide suggestions for further implementation and expansion [36]. Using quantitative and qualitative feedback on the program allows for a more holistic picture of needs, and a logic model provides outcomes to streamline efforts [37]. Evidence supports that the inclusion of appropriate stakeholders, their perspectives, and values, is critically important in this phase of the intervention and to ensure adoption of the program [38]. Additionally, mixed-methods evaluation can be beneficial during implementation, as quantitative methods can be used to develop and test measures appropriate for new conceptual models like the C.L.E.A.R. Program, and qualitative methods can help to understand the context in which the implementation occurs [38]. The Consolidated Framework for Implementation Research (CFIR) was used to guide data collection and analysis to understand the opioid overdose program and implementation factors [39]. The CFIR is a conceptual framework that was developed to guide systematic assessment of multilevel implementation contexts to identify factors that might influence intervention implementation and effectiveness [39]. This established framework is regularly used for systematically analyzing and organizing program implementation determinants and allows for generalizing findings for more immediate application to program implementation. The CFIR framework is also flexible enough to allow other implementation determinants to be incorporated into future program adaptations or tailoring [39], which is helpful when a program needs to evolve over time such as the C.L.E.A.R. Program. This study expands the utility of this framework by combining qualitative and quantitative formative evaluation findings to issue recommendations. Further, combining CFIR with Diffusion of Innovation (DOI), which is a theory used to translate findings into actionable recommendations of targeted and strategic communication about the program to stakeholders [40], ensures the recommendations are appropriate and realistic to those who are ultimately tasked with adoption and implementation of them. The DOI provides a framework for dissemination of information and can be effectively used to promote and implement a public health program [41] given its roots in communication and social science theory.

This evaluation was conducted on the C.L.E.A.R. Program developed in Winthrop, MA, a town of approximately 18,600 people north of Boston. In 2016, Winthrop ranked 17th out of 351 municipalities in MA for opioid-related fatalities per capita (38.9 deaths per 100,000) [42]. Since then, the innovative program has experienced success but also reported gaps in the sharing, collection, integration, and reporting of data across all stakeholders of the program (e.g., public safety, public schools, EMTs, substance use programming, mental health services, community-based organizations). In addition, the collaboration and communication of agencies not yet fully affiliated with the C.L.E.A.R. Program has prevented increased outreach and targeted services, which is more problematic in the COVID-19 context [28] given the need for more collaborative emergency responses, yet overburdened agencies are struggling to respond to needs with available resources. The COVID-19 pandemic has resulted in inconsistent and inadequate federal and state guidelines and communication [43, 44]; a need for increased staff capacity [45]; a lack of data and community partnerships [43]; and, inabilities to respond to other key public health services [46]. In particular, the onset of the pandemic shifted service delivery and care structures leaving those with opioid use disorder more vulnerable and unable to receive care [47]. Accordingly, the C.L.E.A.R. Program was integrated into the town’s public health emergency response through coordinated efforts with a number of stakeholders, creating an opportunity to assess the collaboration of agencies in addressing this issue as the program adapts to the changing landscape.

This study aimed to 1) identify stakeholders for inclusion in formative evaluation, 2) identify facilitators and barriers to adoption and implementation of the program, 3) offer strategies for communication to foster engagement, and, 4) build on the literature for use of a framework to guide evaluation and communication approaches. The formative evaluation of the C.L.E.A.R. Program is a necessary step in identifying barriers to implementation and subsequent recommendations for practice [31] and is warranted given 1) it is an established program but in need of additional collaborators to expand the reach within the community and to other communities; 2) the shifting substance use landscape warrants a further investigation into the facilitators and barriers of accessing services and uncovering gaps in those services; and, 3) there are competing demands on agency’s time and resources to invest in implementation of programs that could be addressed through more strategic communication about the necessity of adopting the program. The aims of the formative evaluation are addressed through the combination of CFIR and DOI to issue realistic and actionable recommendations based on the findings.

## Methods

### Conceptual framework

In order to understand acceptance and implementation of the C.L.E.A.R. Program, mixed methods formative evaluation data collection, analysis, and results were organized according to the CFIR [48], a framework that can be especially helpful to guide in measurement of determinants (i.e., facilitators and barriers) that may impact the implementation of an intervention and can subsequently be addressed in expansions or enhancements to the intervention. The CFIR constructs are organized into five domains and include: characteristics of the intervention; outer setting; inner setting; characteristics of individuals; and process and represent multiple disciplines that influence the implementation of complex, multi-sector programs [48], such as the C.L.E.A.R. Program. The constructs were examined for relevance to the evaluation and were used in the creation of the survey and interview guide and in accordance with CFIR guidance [49]. While all domains were included in the construction of the interview, not all domains were addressed by stakeholders as outlined in the Data Analysis section. For the purposes of this study, “stakeholders” is used to describe “an individual, group, organization, or system who are influenced by or able to influence a project or also defined as an actor or interest group to highlight those individuals or groups who have some role in making a decision or executing a decision.” [50] This study has been reviewed and determined an exempt study by the Boston University Institutional Review Board (IRB #H-42107).

While the CFIR contains a variety of constructs that could ultimately be applied to analysis of data, we utilized a menu of constructs approach [51]. In this approach, the CFIR constructs are pre-determined and used for development of data collection instruments (i.e., interview guides and surveys) thereby focusing the data collection and limiting the time needed for stakeholders to respond. This also allows for inclusion of only those constructs that are most relevant and not the entire framework. While all constructs were represented in the data collection tools, the resulting constructs from CFIR emerged from the qualitative coding as those addressed in interview responses. We examined any remaining constructs not initially coded to determine if those constructs were relevant and would require another round of coding, however, the remaining constructs were deemed not applicable to the interview responses. Accordingly, the data collection instruments for the stakeholder analysis (explained below) were constructed by the research team after the initial identification of priority stakeholders to interview.

The success of a program, especially a multi-sector program, relies on multiple stakeholders moving through a continuum from awareness of the problem, initial use of the innovation or program to address the problem, and ongoing application of the program for sustainability [40] to result in meaningful change. We used DOI [36] to organize the stakeholder findings from CFIR into actionable recommendations for strategic communication for awareness (i.e., promotion), engagement (i.e., implementation), and collaboration (i.e., sustainability) of the C.L.E.A.R. Program. The selection of these three factors for organization of findings and subsequent recommendations aligns with the necessary process for the adoption, initial use, and ongoing sustainability of an intervention, which is particularly applicable to the C.L.E.A.R. Program given the need for communication about the program and the decision-making process for collaboration from multiple sectors to ensure success. Accordingly, the DOI has five attributes that dictate this three-stage process to decision-making for stakeholders and include relative advantage (benefits of an innovation), compatibility (consistency of an innovation with values and needs), complexity (perception of difficulty understanding and using an innovation), trialability (ability to experiment the innovation before adoption), and observability (observation of results of an innovation) [40, 52]. These attributes align with constructs of the CFIR allowing translation of findings into practice.

### Study design

The goal of this mixed methods formative evaluation was to explore implementation of the C.L.E.A.R. Program and the perception of need across stakeholders especially given the shifting context introduced by COVID-19; identify areas for improvement in collaboration, engagement, and communication; examine data needs for comprehensive system-level approaches; and, inform communication strategies to promote the goals and expected outcomes of the C.L.E.A.R. Program internally to stakeholders for collaboration.

**Fig. 1:**
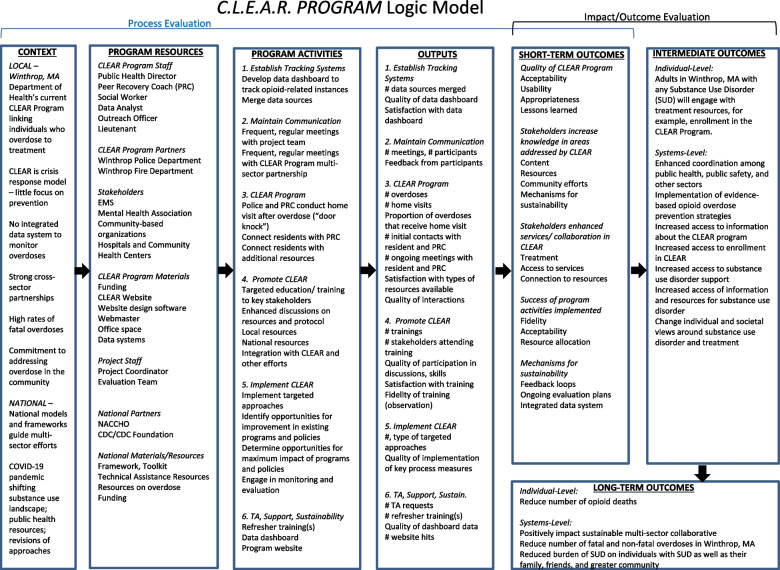
Logic model for a multi-sector community-based opioid overdose program: the C.L.E.A.R. Program

The formative evaluation included an intensive stakeholder analysis to determine the priority individuals to engage in the stakeholder interviews. A survey was administered to assess perspectives, responsibilities, and current C.L.E.A.R. Program collaboration status of each priority stakeholder and was guided by the CFIR domains. The semi-structured interviews were guided by CFIR in addition to the results from the survey and the C.L.E.A.R. Program logic model (Fig. 1) [53]. Results and recommendations for program adoption, implementation, and sustainability are organized by DOI [40].

### Stakeholder analysis

An analysis was performed to determine the stakeholders most relevant to the C.L.E.A.R. Program and therefore targets of the formative evaluation. Stakeholders, defined as an individual or organization influenced by or able to influence the C.L.E.A.R. Program [54] or who have some role in decision-making [55], included both those already invested and those not yet invested in the C.L.E.A.R. Program. The stakeholder analysis followed a stepwise process [54] to both identify the stakeholders and then assess each on factors known to be important to decision-making for collaboration and investment in a program [55]. First, key stakeholders associated with the C.L.E.A.R. Program were identified by the lead program organization’s director in a process which considered who is impacted, who benefits, who controls the resources needed, who influences opinions of each stakeholder, and who can stall progress of the project [54]. Second, the level of power and interest of each stakeholder identified by the director was determined on a 5-point Likert scale (1 = very low, 2 = low, 3 = average, 4 = high, 5 = very high). Interest was assessed by considering “how much is this stakeholder invested in the efforts of the C.L.E.A.R. Program?” and power was assessed by “how much power does this stakeholder have to affect the C.L.E.A.R. Program in a positive or negative way?” [55]. Third, a composite score of power and interest for each stakeholder was calculated by the evaluation team where higher scores indicated high power and interest and therefore were a priority contact. The evaluation team identified stakeholders based on the composite scores that would be ideal to interview, which ensured representation across interest and power rankings with more concentration in high power and interest as the group most influential and most necessary for collaboration [54]. Finally, the director classified each stakeholder according to their stage of change following the Transtheoretical Model [56, 57] and according to how motivated they currently were and whether they were planning to take actionable steps towards the implementation of the program. Following theories of motivation [58], each stakeholders’ perceived needs, benefits, and resistance were further explored with the director to establish a foundation for questions and probes in the interviews.

Fifteen stakeholders were identified through this process – five stakeholders with high interest and high power, eight with low interest and high power, one with low interest and low power, and one with high interest and low power. Stakeholders’ perceived needs (i.e., motivations, specific interests), benefits (i.e., contributions), and resistance (i.e., opposition and barriers) were also assessed during the analysis. Of the 15 identified stakeholders, nine were recommended by the evaluation team for follow-up contact with oversampling of the categories most heavily concentrated by stakeholders (i.e., high interest and high power) and with the most influence [54].

### Study sample

Follow-up contact was recommended to represent agencies that had either high power, high interest, or both (60%, *n* = 9) identified in the stakeholder analysis and represented private and public agencies in Winthrop and the surrounding counties. Some of these agencies were directly involved in substance use prevention efforts, mental health services, and treatment resources and others were more indirectly involved as first responders or youth-serving institutions. The primary contact for the C.L.E.A.R. Program within the agency was determined to be the stakeholder for this evaluation. Of the nine stakeholders contacted, six were available for an interview (66.7%) and eight (88.9%) responded to the survey.

### Data collection

Two primary sources of data collection assessed stakeholder perspectives – close-ended surveys and open-ended semi-structured interviews – and the CFIR was used in the development, data coding, and analysis of both. The survey administered online via Qualtrics in October 2020 was designed to take no more than 15 minutes to complete. The survey assessed respondent/agency characteristics, perceptions and knowledge of the opioid epidemic and the C.L.E.A.R. Program and current and/or past engagement with the C.L.E.A.R. Program. Responses from the survey provided context for the evaluators on the perspectives, knowledge, and engagement with the program, which allowed for tailoring of the interview guide to maximize the interview time by exploring concepts and themes most appropriate to the stakeholders. This resulted in prioritization of interview questions and tailoring of questions through specific probes.

The one-hour semi-structured interview was conducted over Zoom and was developed by the evaluators using the data collected from the surveys in order to explore responses more deeply with qualitative information. The interview concentrated on open-ended questions that focused on four specific areas to gain deeper understanding of the program (described below). Within each area, the content of the questions corresponded to the appropriate CFIR construct. Accordingly, the interview guide was organized by 1) awareness and attitudes (awareness of opioid overdose in the communities served by the agency, knowledge of Winthrop’s C.L.E.A.R. Program, and the agency’s ability to serve the community); 2) collaboration (the agency’s history with and interest in collaborating with opioid overdose programs, and the benefits and barriers to those collaborations); 3) engagement (factors that foster agency connection with opioid overdose programs); and, 4) communication (successful outcomes of collaboration and communication of the outcomes as well as supports needed for sustainability). Each section of the interview used probes as appropriate from the survey results and aligned with CFIR constructs (Table 2) to qualitatively explore the four areas of exploration.

### Data analysis

Surveys were analyzed in the survey software system (Qualtrics, Boston) using descriptive statistics to examine continuous and categorical data. Interviews were designed, conducted, and analyzed in accordance with the consolidated criteria for reporting qualitative research (CORE-Q) [59]. The evaluation team consisted of two evaluation staff who had previously been trained on qualitative coding. The team jointly conducted the interviews. The team independently coded the transcripts using the framework as a guide for categorizing interview findings into constructs. The team met to compare coding of transcripts and discuss differences in coding and interpretation. This process continued for each transcript and all differences were resolved by consensus. To analyze the stakeholder interviews, a content analysis [60] with deductive approach [51] was used with the pre-determined CFIR domains and constructs used as the coding process. If other constructs and domains emerged in the analysis inductively then they were included in the final results (for example the characteristics of individuals domain, which initially was not a point of focus for the evaluation). All constructs were represented except trialability, individual stage of change, and formally appointed internal implementation leaders. Since the interviews were conducted as semi-structured with an open-ended format, interview responses were examined for inclusion into any of the applicable CFIR constructs in the interview guide. This allowed for inclusion of comments and responses in the analysis that were made by the stakeholder through a more narrative approach to the interview rather than direct question and answer.

## Results

### Survey findings

There were a total of eight survey responses with seven different agencies represented (one agency had two respondents). Stakeholder respondents represented public safety (37.5%), public health (25%), behavioral health (12.5%), community-based agency (12.5%), and public schools (12.5%). Job responsibilities ranged from agency leadership (24%), advocacy (12%), public information (12%), community health (8%), health educator (8%), preparedness (8%), office/administrative (8%), and other (20%) with half of respondents (*n* = 4) serving at their agency for more than ten years and none serving less than four years. Funding sources varied for each agency with the majority coming from federal sources (25%), local sources (20.8%), and state sources (20.8%) (*data not shown*).

The majority of respondents strongly agreed there is an opioid epidemic nationally (87.5%) and that there is an associated burden of the opioid epidemic within Massachusetts (87.5%) and within Winthrop (62.5%). In addition, most strongly agree that responding to the epidemic requires more resources than are currently allocated (87.5%). Half of respondents (50%) report that the protocol in their community for responding to the opioid epidemic needs to be updated or better communicated with 50% reporting it is in a steady state and 50% reporting it is in a resurgence. Agencies reported using a variety of resources to respond to the epidemic including state resources (87.5%, *n* = 7), local resources (75%, *n* = 6), informal discussions/meetings on the opioid epidemic (75%, *n* = 6), training on the opioid epidemic (62.5%, *n* = 5), discussion forums/meetings on the opioid epidemic (i.e., agency internal meetings, community town halls, etc.) (50%, *n* = 4), and technology/tracking systems (50%, *n* = 4).

More than half (63%, *n* = 5) of agencies were somewhat familiar with the C.L.E.A.R. Program, 25% (*n* = 2) were very familiar with the program, and 12% (*n* = 1) were not that familiar with the program. Four respondents (50%) reported that they don’t currently collaborate with the program but have in the past, two (25%) reported that they do not currently collaborate nor have they in the past, one (13%) reported currently collaborating with the program, and one reported being unsure (13%). Of the seven agencies that do not currently collaborate with the C.L.E.A.R. Program or who are unsure, 66% are extremely likely and 34% are moderately likely to collaborate in the future.

Agency collaborators have offered services as regional partners, service providers, through the justice system, or by providing resources to the program. Among those agencies that currently collaborate or have collaborated with the C.L.E.A.R. Program there is agreement that the leadership is knowledgeable, collaborative, and that there is a strong need for the C.L.E.A.R. Program. There was strong agreement in continuing to collaborate with the program (Table 1).

**Table 1:** Survey questions for collaborating (*n*=5) and non-collaborating (*n*=3) agencies, by stage of diffusion of innovation

Diffusion of Innovation Stage	Collaborating Agencies (*n*=5) Question % agree (n)	Non-Collaborating Agencies (*n*=3) Question % agree (n)
Use, Collaboration Engagement, Continuation	The leadership in the Winthrop CLEAR Program was collaborative 60% (3)	---
Use, Collaboration Engagement, Continuation	The leadership in the Winthrop C.L.E.A.R. Program was knowledgeable 80% (4)	---
Awareness, Adoption Use, Collaboration	The Winthrop C.L.E.A.R. Program added value to the efforts of my agency in responding to the opioid epidemic 40% (2)	The Winthrop C.L.E.A.R. Program would add value to the efforts of my agency in responding to the opioid epidemic 100% (3)
Awareness, Adoption	I think there is a strong need for the Winthrop C.L.E.A.R. Program in responding to the opioid epidemic 80% (4)	I think there is a strong need for the Winthrop C.L.E.A.R. Program in responding to the opioid epidemic 100% (3)
Awareness, Adoption	Others in my agency think there is a strong need for the Winthrop C.L.E.A.R. Program in responding to the opioid epidemic 20% (1)	Others in my agency think there is a strong need for the Winthrop C.L.E.A.R. Program in responding to the opioid epidemic 66.67% (2)
Use, Collaboration Engagement, Continuation	The Winthrop C.L.E.A.R. Program collaboration fills gaps in skills in my agency to address the opioid epidemic 20% (1)	The Winthrop C.L.E.A.R. Program collaboration would fill gaps in skills in my agency to address the opioid epidemic 100% (3)
Awareness, Adoption Use, Collaboration Engagement, Continuation	The Winthrop C.L.E.A.R. Program collaboration fits well within the existing structure of my agency 20% (1)	The Winthrop C.L.E.A.R. Program collaboration would fit well within the existing structure of my agency 100% (3)
Use, Collaboration Engagement, Continuation	I would collaborate with and/or continue to collaborate with the Winthrop C.L.E.A.R. Program to address the opioid epidemic 100% (5)	---

Of the three agencies that have not collaborated with the C.L.E.A.R. Program or are unsure, all (100%) reported strengthened ties to the community, streamlined data systems for tracking and outreach, building trust within the community, and building capacity within the community as possible benefits. Resources they would find helpful in collaborating included reports with both qualitative and quantitative data (37.5%, *n* = 3), newsletters (12.5%, *n* = 1), and toolkits (12.5%, *n* = 1).

Across all stakeholders, future collaborations included sharing data for tracking and outcomes, partnering on funding opportunities, and promotion of the program (i.e., shared newsletter, flyer, social media, etc.). Resources to foster ongoing collaboration with the program included flyers, memorandums of agreement, social media, reports, infographics, websites, newsletters, and toolkits. All agencies reported that the C.L.E.A.R. Program adds value to the efforts of their agency, fills gaps in their agency, and fits within the structure of their agency, and all agreed there is a strong need for the program (Table 1).

Respondents were asked about communication channels used within their community for emergency efforts and reported Facebook (75%), email (63%), text messaging (50%), print media (38%), automated phone calling (38%), and a hotline or call center (25%). These findings are important to consider in making recommendations for communicating about the C.L.E.A.R. Program.

### Interview findings

**Fig. 2:**
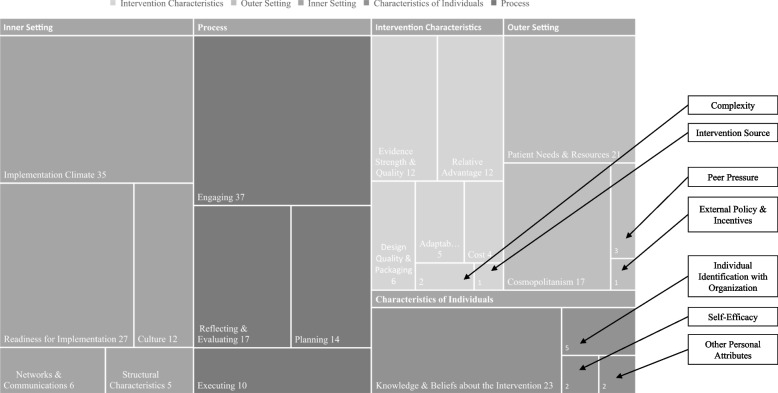
interview findings for collaborating (*n* = 5) and non-collaborating (*n* = 2) agencies, CFIR constructs by coding density

Six of the eight survey respondents were interviewed with one respondent not available. The qualitative interview findings are presented by CFIR constructs and summarized into phases aligning with DOI for practical application. For each of the five CFIR domains (characteristics of individuals, intervention characteristics, process, inner setting, outer setting) the hierarchy of inner constructs that comprise the domains (i.e., implementation climate, engaging, relative advantage, patient needs & resources, knowledge & beliefs about the intervention, etc.) are depicted with the size of the box indicating the number of times a construct was referenced in the interviews. For example, implementation climate is the most densely coded construct in the inner setting domain with 35 codes, which is demonstrated by the larger surface area of that box (Fig. 2) indicating this was a substantial area of focus in the interviews. These are presented and interpreted alongside the results by domain below. Ideas related to the construct, themes and relevant quotes are provided in Table 2.

Actual and perceived barriers such as cost, evidence strength, resources, patient needs, external policies, and champions, are reflected in the responses. In addition to the constructs presented in Fig. 2 the following subconstructs were coded: tension for change, compatibility, relative priority, organizational incentives and rewards, goals and feedback, learning climate, leadership engagement, available resources, access to knowledge and information, opinion leaders, champions, and external change agents. All illustrative quotes are provided by unique respondents.

### Domain 1: Intervention Characteristics

This domain addresses factors that influence whether the intervention is implemented successfully. While this domain can include factors and components specific to the intervention that are actual barriers or facilitators including cost and evidence strength. It largely addresses perception of the factors influencing an intervention’s successful implementation, which includes the decision to adopt given the idea is perceived as new, innovative, and/or necessary. Evidence strength and quality and relative advantage had the strongest coding densities (Fig. 2) with stakeholders considering the degree to which the C.L.E.A.R. Program is better than an alternative (Table 2), which is a factor in considering motivations for adoption [40]. For example, stakeholders were aware of C.L.E.A.R. Program activities and believe there were gaps in the services provided since program efforts are concentrated in a small geographic area even though surrounding communities struggle with opioid overdose and lack the public health infrastructure needed to address the problem. According to one respondent (Table 2): *[A] clear gap is safe locations for people to use drugs. C.L.E.A.R. Program delivers naloxone. Gap would be working closer with folks who are abstinence-minded, very hard line about recovery, maybe some antiquated views about naloxone distribution to address abuse. It [naloxone use] just needs to be normalized*.

**Table 2:** Qualitative summary of stakeholder interviews (*n*=6) by consolidated framework for implementation research domains and constructs

CFIR Domain^**a,b**^				
Intervention Characteristics	Outer Setting	Inner Setting	Characteristics of Individuals	Process
***Intervention Source*** […] is familiar that it [overdoses] is happening more often than they would like it to in the community. […] is aware of the response team dealing with the issues at hand, police department in general or outreach after it. ***Relative Advantage*** Did work for a while trying to outreach with the initial recovery coaches […] but felt Winthrop had a fair amount of resources so didn’t continue to provide support. I like what they are trying to do but […] doesn’t necessarily agree with the model. […] I don’t see how people want to open their door to cops. “[Peer recovery coach] should also be a part of the door knock process – friendly face in plain clothes of ‘I’ve been there, done that,’ opens the door to bigger conversations.” ***Adaptability*** Clear gap is safe locations for people to use drugs. C.L.E.A.R. Program delivers naloxone. Gap would be working closer with folks who are abstinence-minded, very hard line about recovery, maybe some antiquated views about naloxone distribution to address abuse. It [naloxone use] just needs to be normalized. Gaps that C.L.E.A.R. can provide: Ability to share data when overdoses occur, perform post-overdose outreach, being able to respond in 24-hour timeframe… ***Evidence Strength & Quality*** Amount of calls decreasing. They have a number of communities that have seen a decrease in opioid overdose, decrease in Narcan and patient touches have gone down. Some communities haven’t had an impact on the issue at all because they don’t have infrastructure. Addicts are still using but there aren’t increases yet related to COVID. One concern with data is that one individual is counted for each instance even though same individual. If that individual passes away the data goes down the next month. ***Complexity*** History [pre-dates the current leadership team] that Winthrop doesn’t want to collaborate with outside agencies. Some of that is why agency doesn’t collaborate and some of it is due to COVID.	***External Policy & Incentives*** They say this [not enough female detox places] is because there aren’t beds. The way this was modeled it’s geared towards men. There is no trauma specific programming to females... the caretaker piece, women can’t find people to watch their kids and they don’t get tailored services so they leave or don’t show up. ***Patient Needs & Resources*** Winthrop has a much bigger alcohol abuse problem than it does opiates. There is a rise in cannabis overdoses across entire state as well… Think beyond the idea of opiates. Need the same infrastructure, opiates or not. The system and the process will be the same for any substance and the stakeholders are all the same. “Are we doing anything with that person to be proactive, before a kid goes out and overdoses. What are we doing proactively and how are we doing reactively?” ***Peer Pressure*** [Other town] is a great example – any type of overdose they flag that call and send that report to a group of stakeholders – police, fire, mental health. They then take the report, identify the case, and make arrangements to door knock and track the individual. “This has been really effective to reduce rates [of overdose].” ***Cosmopolitanism*** [It’s important to talk to the leadership] for C.L.E.A.R. Program to see what info they want. If they want pre-hospital EMS report so they can get the complete report, inclusive of paramedics and EMS conversations. They can get more context other than just law enforcement reports. Not broached […] as an opportunity until recently. [The current partners] “are a tight group [Winthrop Public Schools, Public Health, and Public Safety] so the confidentiality among us is very strong……pact between us that allows sharing of information.” Knowing each other separates us from the average town or city collaboration. We all live in the community so that’s another level of understanding [and passion]. “Winthrop is an island all to itself. They are very cut-off and secluded from everything else. And I think they [may] like it that way.”	***Structural Characteristics*** The goal is that there would be an entire unit led by [a trained officer] from each “house” to handle cases. Other officers are already in touch to replace [the primary officer] in situations where he’s busy, more often with a Behavioral Health (BH) incident and less likely in an overdose. This is informal, but the process could be formalized. In BH there is lots of recidivism (less paperwork). ***Networks and Communications*** We […] need to communicate more – we used to meet [regularly] and now we don’t. “How do we communicate more efficiently and effectively? Share the lows instead of keeping it all for ourselves?” Personality and egos can be challenging – local government is a funny thing. Different agendas and different ideas/level of knowledge. It [can] impact the level of collaboration across the board. Collaboration with the program – “it doesn’t matter where you are from it makes more sense to build capacity across the board”. Staffing [can also make it] hard to collaborate. ***Culture*** [Certain people have] tried to break down the stigma a bit but I don’t know if the culture has shifted. “It’s a huge undertaking…it’s the town culture, not a department of public health culture” ***Implementation Climate*** I would love to collaborate with [other mental health-focused agencies] but I don’t [know if it] will happen. My [peer recovery] coaches used to be over there and there was a good relationship. But over time it’s waned. ***Implementation Climate*** It shouldn’t be always up to our harm reduction program to meet gaps because we lack resources or partners to meet them. [Other towns] have substantial funding and resources to tie and work closely together. ***Readiness for Implementation*** Each community has its own strategic plan… […] wants to look at all these overarching strategic programs across all communities. Their strategic plan may look different than what Winthrop has in place. Need to sit down and identify what are collective goals?	***Self-efficacy*** “If I lock someone up for 18 months, then the crime rate drops for 18 months. If we’re able to turn someone’s life around, then the crime rate drops for 30 years…” ***Other Personal Attributes*** [What outcomes matter most to you?] Wraparound piece and education of family. If someone is using, they are extremely unhealthy and that sends a ripple effect to everyone else. “What resources are out there and how is that affecting everyone else so you can protect yourself and the person you love?” ***Individual Identification with Organization*** [The program should] reach out to the people they need on board. [Message should be] we want to enhance the program we have now. We are doing a re-launch and we need your support and really looking to collaborate… “level of authenticity” things seem one way but are actually another. Own what you didn’t do – “We see a lot of smoke and mirrors in public health... it’s not a failure it’s a learning opportunity”. ***Knowledge & Beliefs about*** ***the Intervention*** Right now Winthrop [to my knowledge] is the only one without a naloxone program (OEND program). So when overdose occurs it’s not just about providing access to recovery but looking at stages of change and meeting them where they are – if they want to use then [the program should] help them use in safety. Help [those in need] build up readiness to get to the next level. This is a big gap that law enforcement can’t do… As it [The C.L.E.A.R. Program] evolved and went into different staffing it was a complicated process. “It’s a very new field in itself…not a lot of standards and best practices to implement and supervise coaches effectively…”. Went through number of trial and error and how to best supervise – supervisor coach. The supervision wasn’t working out.	***Planning*** Pursue larger opportunities for funding. [In order to do this] need to have institutional knowledge and infrastructure to pursue larger opportunities. Winthrop is an important partner – “need to have the relationship to feel confident so we can pursue [additional funding].” Quarterly meeting would be great. Recovery support opening near Winthrop and co-occurring in-house that also borders. […] not sure if Winthrop knows about these resources. “If we met more often and could be on the same page it could be helpful and beneficial.” ***Executing*** What is happening with an individual 6 months from now? [There is a need for] Tracking individuals long-term to observe recidivism, identify gaps in programming These [Ex. recovery support] are some things they could implement on a local level but would be better to also work with Winthrop to share and pool resources, shared goals, and then streamline data. ***Engaging*** Shared data is so important, but level of buy-in from leadership is more important for sustainability. In person meeting with the goal of coming together and relaunching the program and re-establishing goals ***Reflecting & Evaluating*** Shared information system that is secure – the right people are receiving the right information and receiving the right access that’s really important… “Some type of platform, that we can all go in and have access to… Success stories, where are they at”

^a^ Quotes are presented as stated unless edited to remove identifying information as indicated by […] in the quote. The use of brackets is also used to insert clarifying information not part of the response and/or quote

^b^ Some quotes were removed to protect stakeholder confidentiality and instead general themes are provided

### Domain 2: Outer Setting

This domain addresses external factors impacting the intervention such as agency networks, communication, policies and incentives, barriers and facilitators to meeting needs of the population, and competitive pressure to implement an intervention [48]. Patient needs and resources had the strongest coding density (Fig. 2) with themes emerging that impact trialability [40], a component of DOI that results in testing the intervention to determine if it meets the needs given available resources. A common theme observed across stakeholders was that the C.L.E.A.R. Program has gaps in services around prevention and that this provides an opportunity for program enhancement to further implementation (Table 2). Additionally, some reported the existing partnership between public safety and public health was very strong, which enhanced their ability to implement interventions successfully, but that made it difficult for those not currently collaborating with the C.L.E.A.R Program or servicing townships outside of Winthrop to collaborate; For example (Table 2), *[The current partners] “are a tight group [Winthrop Public Schools, Public Health, and Public Safety] so the confidentiality among us is very strong……pact between us that allows sharing of information.” Knowing each other separates us from the average town or city collaboration. We all live in the community so that’s another level of understanding [and passion]*.

### Domain 3: Inner Setting

This domain addresses internal factors of an organization that impact the implementation of an intervention including the internal communication, norms and values, structure and system, capacity for change and receptivity of individuals, and commitment within the agency to the intervention [48]. In this study, the agency’s commitment to accept (adoption) and capacity to use (implementation) the C.L.E.A.R. Program aligns with the first two stages of DOI.The most important construct was implementation climate (Fig. 2) with stakeholders considering the structure and resources necessary to support the program as well as the importance of external communication to sustain collaboration and enhance existing structures beyond Winthrop. As part of this external communication, they identified stigma around opioid use in the community as a barrier to successful implementation with one stakeholder commenting (Table 2) that *[Certain people have] tried to break down the stigma a bit but I don’t know if the culture has shifted. “It’s a huge undertaking…it’s the town culture, not a department of public health culture”*.

### Domain 4: Characteristics of Individuals

This domain addresses the thoughts, perceptions, knowledge, confidence, beliefs, and commitment to change of individuals that comprise the organization [48]. Ultimately, the commitment to, or implementation of, an intervention is held by individuals; their personal attributes can substantially impact implementation regardless of their agency’s commitment to its success. This is essential when considering different adopter categories as early adopters of a behavior change have different characteristics [40] than those who adopt the behavior later [61]. In this study, knowledge and beliefs about the C.L.E.A.R. Program had the strongest coding density (Fig. 2) as stakeholders considered their previous experience with the program and gaps in the C.L.E.A.R Program observed through lack of a naloxone program. According to one stakeholder (Table 2), *Right now Winthrop [to my knowledge] is the only one without a naloxone program (OEND program). So when overdose occurs it’s not just about providing access to recovery but looking at stages of change and meeting them where they are – if they want to use then [the program should] help them use in safety. Help [those in need] build up readiness to get to the next level. This is a big gap that law enforcement can’t do…* They also identified a need for clearer communication to stakeholders and community partners to enhance collaboration and program reach.

### Domain 5: Process

This domain addresses implementation and sustainability through consideration of engagement strategies (i.e., education, marketing, training), established plans for implementing the intervention, delivery of the intervention according to the established plan, and communication and feedback about the intervention and its implementation. Observability, or the opportunity to see the intervention in practice, can prompt ongoing use [40]. In the evaluation of the C.L.E.A.R. Program, engagement had the strongest coding density (Fig. 2) with stakeholders commenting on the need for data feedback and shared goal-setting. Stakeholders expressed their need for increased funding to improve infrastructure, better data tracking mechanisms that allow for secure data sharing, and leadership buy-in that could be improved by strategic communication with one stakeholder commenting (Table 2), *“Some type of platform, that we can all go in and have access to… Success stories, where are they at”*.

The findings presented are specific to the context and program within Winthrop and will be used for revision and improvement of program components, new and sustained engagement of collaborators, adaptations and enhancements given the shifting substance use landscape, and determination of resource allocation particularly with the increased need yet taxed public health and public safety infrastructure. The findings, however, provide valuable insights to other communities that are planning or currently implementing substance use programming and offer insights to the evolving nature of a multi-sector program that relies on high quality collaboration and communication to effectively meet objectives and respond to shifting needs of its recipients in a resource-constrained environment.

## Discussion

This mixed-methods formative evaluation aimed to uncover how existing and future stakeholders could be leveraged and more fully engaged to further the reach and efforts of an opioid overdose program at a time when expansion and enhancements are necessary to meet the shifts resulting from COVID-19. This study found that stakeholders agree that the work of the C.L.E.A.R. Program is important and that the public health and public safety relationship is strong, but there are crucial gaps in the services provided such as prevention efforts and safe injection sites (Table 2). The study also found the overall infrastructure such as internal and external communication, data tracking and sharing, funding and leadership buy-in of the program could be enhanced, as these are factors with demonstrated importance to the speed and success of program adoption [62]. Consistent with recommendations for effective dissemination of substance use programs, the need for collaboration and expansion of services into the surrounding communities was reported as integral to the reduction of opioid overdoses [63] and tailored communication of the program to the underserved populations essential to sustainability [64].

Limited evidence on the design, implementation, and evaluation of community-based opioid-related programs exist [5] and methods to strategically promote programs for sustainability are even more limited [63]. Those that do exist demonstrate the need for more formative evaluation to best address barriers and facilitators to implementation [5] and an understanding of context for expansion to other settings [65]. Formative evaluation is well established as a mechanism for understanding perceived and actual barriers to implementation and is not only necessary to translate findings into actionable recommendations [31] but allows for application of findings during the study to assist implementation teams on immediate adaptations and enhancements to the program in order to be most effective in practice [66]. Using both qualitative and quantitative data collection strategies can result in a rich and thorough investigation into an intervention that results in increased understanding and uptake of it [38, 67].

Conducting a formative mixed methods evaluation of the C.L.E.A.R. Program allowed insight to the implementation of the program as designed but also as it was being adapted to fit the COVID context [38]. In particular, quantitative findings showed that identified collaborators for the program – both existing and new – recognized the need for opioid overdose programming, valued the multi-sector approach to addressing the problem, and believed their involvement was essential to responding with a comprehensive and coordinated effort but that many perceived and actual barriers focused on lack of communication around the shared goals of the program, the process of data tracking and information sharing, and the specific role of each agency in the existing design of the program and adaptations to it. Accordingly, and consistent with other research on substance use program implementation [68] and specifically in clinical settings [68, 69], the CFIR guided the evaluation to identify gaps where strategic communication could enhance the multi-sector community-based C.L.E.A.R. Program and the DOI provided a foundation for recommendations for communications that consider the continuum from adoption to implementation to sustainability [70] with recommendations grounded in evidence-based implementation strategies [71].

Areas of improvement in the design and implementation of the C.L.E.A.R. Program align with the CFIR and strategies to address those areas can be organized according to the DOI.Within the intervention characteristics domain there were reported gaps in the program to meet the needs of a larger geographic area. Redesigning the program means examining the current infrastructure of the collaborating agencies to determine how they provide support to the program or identify the enhancements needed [63], particularly in the changing landscape from COVID-19, and is especially important for adoption across collaborators [62]. In addition, findings from the outer setting domain identified gaps in community-based prevention-focused activities both within the design of the C.L.E.A.R. Program and its connection to ongoing prevention activities. Identifying agencies and efforts that fill these gaps and promoting the importance of collaboration of these agencies in a more unified, better-resourced approach results in more widescale adoption and more effective and comprehensive program implementation [63]. Additionally, we mapped the feedback received from the stakeholder interviews to the list of 73 evidence-based implementation strategies from the Expert Recommendations for Implementation Change (ERIC) group [71], which has previously been linked to DOI [31] and is described within the three DOI phases below. We ground our findings in those most appropriate to the adoption and implementation of the C.L.E.A.R. Program to issue specific recommendations.

Within the inner setting domain, it was reported that effective collaboration entailed prioritizing external communication with partners not just within the immediate catchment area of the program but in areas that affect both implementation and the stigma that surrounds opioid use. Communication encouraging collaboration with the C.L.E.A.R. Program requires careful messaging to the target population and stakeholder agencies. This messaging should be pre-tested to ensure sensitivity and that it fits the context [72].

Gaps uncovered in the characteristics of the individuals domain can be addressed through communication focused on sustainability that is appropriate, consistent, and tailored to the program’s success, challenges, and areas of expansion. This is important for stakeholder investment in both their agency’s long-term engagement of the program but also in understanding the importance of their individual contribution to successful outcomes [73]. This can be achieved by highlighting the values and activities of the individual and its agency on their direct contribution to the success of the program and the ways they can contribute longer-term [64]. Additionally, findings from the process domain validate the use of data, testimonials, and results from evaluation activities to foster ongoing engagement in the C.L.E.A.R. Program. Real-time data dashboards and data systems can help invested agencies witness immediate impact as well as identify areas for ongoing improvement. These findings can be communicated through program leadership and should be continually evaluated to ensure appropriate diffusion [74].

As outlined, considering implementation determinants organized by CFIR domains [48] results in practical strategies that align with the stages of DOI [40]. These are essential to consider when tailoring communication efforts to stakeholders and eventually the community served [41, 75] particularly for substance use prevention [63] during a time of transition or changing context [76] such as with the COVID-19 pandemic. Further, it is difficult to plan for sustainability without considering how stakeholders implement the program, the processes they follow for feedback and dissemination of findings, and the existing structure and resources of their agency.

**Fig. 3:**
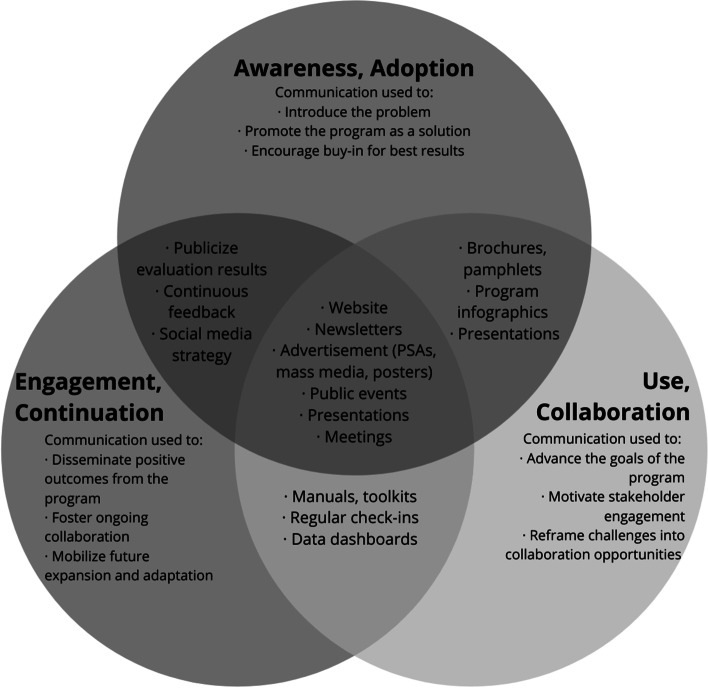
Communication strategies for stakeholder engagement, use, and continuation of a multi-sector community-based program

Effective communication relies on a target population’s ability to receive communication and for that communication to affect some sort of change, whether through adoption of a new behavior, uptake of new information, trialability of a new program, or commitment to ongoing implementation [40]. Information disseminated should increase knowledge while also considering the target of the behavior change (i.e., individuals, agency stakeholders) to provide concrete strategies that translate the information into practice [77]. To that end, engaging stakeholders early allows for a thorough understanding of the target population, whether program recipients or collaborators, to inform messaging and communication. Specific strategies for the C.L.E.A.R. Program or other multi-sector, community-based opioid overdose programs can be utilized to promote awareness and adoption, to encourage use and collaboration, and to foster engagement and continuation (Fig. 3) [48] and can be further operationalized by considering implementation strategies and the actions needed to address them [71]. The recommendations provided herein focus on dissemination strategies focused on raising awareness of the problem and program for stakeholder engagement and buy-in though we offer suggestions for opportunities for ongoing discourse and conversation in the information disseminated [78].

### Program awareness and adoption through communication

Program advertising through various communication efforts raises awareness of the program for program recipients and stakeholders that make the program stronger and align with implementation strategies focused on communication including developing educational materials, distributing educational materials, tailoring strategies, and using mass media [71] or implementation strategies focused on institutional adoption including identifying and preparing champions, identifying early adopters, and conducting educational meetings [71]. Appealing to individual cognitive factors through targeted communication such as presentations about the benefits of the program and ongoing meetings with stakeholders ensures the right stakeholders, including program champions and early adopters, are involved with these types of programs, leading to use and collaboration (Fig. 3). For example, to create awareness of the C.L.E.A.R. Program among people at risk of opioid overdose, appealing and engaging infographics could be displayed in community spaces where people struggling with addiction and overdose are often present such as in emergency rooms. In addition, use of take-home brochures or pamphlets can lead to increased access to the information at a later time. Finally, in-person discussions and presentations not only allow people struggling with addiction and overdose the opportunity to ask questions and get targeted resources but also offer an opportunity for promotion of the program to stakeholders to address barriers and result in buy-in (Fig. 3).

### Use and collaboration through communication

Utilizing strategic communication with both new and existing stakeholders to introduce and increase multi-sector collaboration is important to programs such as the C.L.E.A.R. Program so the varying needs of the target population can be addressed through availability of a variety of appropriate recovery services [5, 79]. In particular, stakeholders reported that the C.L.E.A.R. Program needs to enhance existing, and forge new collaborations that extend beyond Winthrop to be most effective in addressing new challenges emerging from COVID-19. These collaborations should focus on strengthening internal and external communications, sharing lessons learned and areas for expansion, evaluating efforts, and creating shared data systems. Implementation strategies that align with this feedback and can result in actionable recommendations include accessing new funding, assessing for readiness and identifying barriers and facilitators, building a coalition, capturing and sharing local knowledge, providing ongoing consultation, providing local technical assistance, and using data experts [71]. For agencies primarily providing financial and staffing resources to the C.L.E.A.R. Program, effective communication entails regular meetings with clearly defined agenda items such as identifying funding or prioritizing activities, continued conversations around goal-setting, involvement of experts and stakeholders from other successful implementations, and data dashboards to monitor progress toward outcomes. For agencies that are primarily invested in direct service activities such as providing treatment services, effective communication entails participant testimonials and feedback of program impact to share knowledge, manuals and toolkits outlining the coordination of the service delivery model for best practices in certain contexts, and regular check-ins and technical assistance opportunities to support implementation and address barriers during implementation [80]. Considering implementation strategies such as the above that extend beyond information dissemination and into providing supports, changing structures and systems, and offering opportunities for application can lead to positive outcomes for agency stakeholders that are not achievable with knowledge dissemination alone [81].

### Engagement and continuation through communication

The sustainability of a program is key to addressing a public health problem and is the last stage in the diffusion process [40]. Ongoing engagement of all stakeholders is important so there is continued interest in the program – both to support and use the program. Constant evolution and improvement of the C.L.E.A.R. Program is important so stakeholders and program participants continue to benefit, particularly as the CFIR domains impacting individual perception and external influences were most coded in this study and are consistently important in the field [48]. Implementation strategies to consider in this phase result in a structure that supports ongoing successful implementation and include auditing and providing feedback, developing a formal implementation blueprint, developing and implementing tools for quality monitoring, reexamining the implementation, revising professional roles, and promoting adaptability [71]. For example, in the C.L.E.A.R. Program, this could be accomplished by publicizing results from evaluations through newsletters, media engagement, and a targeted social media strategy with an emphasis on accomplishment of outcomes, lessons learned, and opportunities for adaptation and expansion. In addition, efforts to mobilize future expansion, adaptation, and continued collaboration can be achieved through continuous feedback mechanisms and public events, promotions, and presentations to outline the program benefits and successes, which will be especially important as the post-COVID-19 landscape demands increased access to availability of these types of programs (Fig. 3). Finally, developing plans for long-term engagement and accountability of stakeholders through program planning, resource allocation, and clearly defined agency responsibilities can lead to sustainability of efforts.

Tailored communication to stakeholders and the community is used to 1) create awareness of the program to people who could benefit from it and organizations that will strengthen it, 2) foster collaboration between the established program and meaningful stakeholders, and 3) engage the community and stakeholders on an ongoing basis to move the program forward [75]. Aligning communication strategically to address gaps in adoption and implementation aids in moving the program goals forward and is an antidote to barriers of program use [75] for all stakeholders. For example, a tailored presentation outlining the benefits not only to the community, but to the organization could help prompt a mental health organization hesitant about a partnership with the C.L.E.A.R. Program to engage, thereby increasing resources to people at risk of opioid overdose [5]. Regardless of dissemination strategy, information should appeal to the target population, open opportunities to scale-up the program, and ensure the knowledge is translated into practice [78].

### Strengths and limitations

Our study demonstrates the use of the CFIR to identify strengths and gaps in an existing opioid overdose program and opportunities for expansion and improvement particularly as the post-COVID-19 public health field is shifting. The combination of the DOI to inform strategic communication to address the gaps and promote the strengths for stakeholder adoption, program implementation, and multi-sector sustainability further enhances the findings and recommendations of this study. In addition, the study utilized methodology that focused on mixed methods to inform immediate and sustainable changes [36, 82] from multiple perspectives, allowed for rigorous coding of the CFIR, and examined a variety of communication strategies according to the DOI that while recommended for this particular program, are applicable to other public health programs. The study included a variety of stakeholders representing multiple agencies that both collaborated and did not collaborate with the C.L.E.A.R. Program allowing for a more comprehensive perspective.

Although DOI has been widely used in the public health field [40], much of the evidence for DOI did not originate in public health and does not consider an individual's resources or social support to adopt the new behavior or innovation [48]. Additionally, the C.L.E.A.R. Program that informs these recommendations is tailored to overdose and addiction in Winthrop, MA and other cities and towns may need to specifically tailor communication strategies in their overdose programs to the needs of their communities. While efforts were made to select individuals with power and interest in the C.L.E.A.R. Program, these agency stakeholders may not have representative views of all staff working within the agencies. The small sample size for both the survey and interviews across and within agencies, as well as the lack of use of validated measures, limits the generalizability of the specific findings though offers insight to broader contextual issues and barriers. Additionally, there is limited evidence supporting the effectiveness of the C.L.E.A.R. Program and while it may be a promising approach, additional process and outcome evaluation is warranted to assess its effectiveness and ultimately inform implementation. Lastly, while we did access a variety of perspectives, the community stakeholders represented in our sample did not identify as people who use drugs and reflect the opinions of those working in organizations who support (or could support in the future) the work of the C.L.E.A.R. Program. Future research in this area should address these limitations in the design and conduct of the study.

## Conclusion

This mixed methods study builds on existing literature by exploring the facilitators and barriers to a multi-sector community-based opioid overdose prevention program through use CFIR to systematically identify the factors impacting implementation and DOI to recommend communication strategies to ensure engagement, use, and continuation of the program. With existing programs, such as the C.L.E.A.R. Program, formative evaluation findings can result in necessary adaptations that ensure more widescale adoption and sustainability. This is particularly important when the context around an existing intervention change resulting in shifts in resources, priorities, and stakeholders. This is emphasized with the already severe opioid epidemic being exacerbated by the COVID-19 pandemic.

Specifically, this study found crucial gaps in the services offered and catchment area reached by the C.L.E.A.R. Program but that existing resources and agencies could fill those gaps if appropriately engaged. Additionally, with ongoing collaboration from these agencies, the overall infrastructure of the existing program could be enhanced leading to expansion and sustainability. These findings were further explored through tangible recommendations on ways to communicate to all stakeholders – collaborating agencies and program targets – depending on whether the stage of diffusion was adoption, implementation, or sustainability. While this study explored multiple perspectives in this arena, future work in the implementation and evaluation of community-based opioid prevention programs should consider patient-centered perspectives and needs and how those needs change over time. This will allow continuous redevelopment and revision of substance use programs and policies that aim to address individual needs through agency cross-collaboration leading to targeted, sustainable approaches that improve outcomes for those who are most at risk of opioid overdose.

## Supplementary Information

**Additional file 1.**

## Data Availability

The interviews and data for this study are not publicly available due to privacy, confidentiality, and anonymity considerations, but data that support the findings of this study are available from the corresponding author upon request.

## Abbreviations

C.L.E.A.R.: Community and Law Enforcement Assisted Recovery
CFIR: Consolidated Framework for Implementation Research
DOI: Diffusion of Innovation Theory
