# Supplementary material for: Using formative evaluation of a community-based opioid overdose prevention program to inform strategic communication for adoption, implementation, and sustainability
Source: BMC Public Health. 2023 Feb 15;23:341. doi: 10.1186/s12889-023-15229-2 (PMC9930042; doi:10.1186/s12889-023-15229-2)
Supplement: Supplementary file 1 — Additional file 1. [file 12889_2023_15229_MOESM1_ESM.pdf]

Date: _______________ Participant: _____________ Interviewer: _______________

C.L.E.A.R. Program Evaluation

Stakeholder Interview

(NOTE: The interview questions and probes will be refined prior to the interview using the stakeholder analysis document and the stakeholder survey. The questions will be prioritized to fit within the timeframe of the interview (ideally 60 minutes unless the agency indicated a shorter period of time). Some notes are made in parentheses that indicate the related Consolidated Framework for Implementation Research (CFIR) construct and are not to be read to the participant.)

Introduction

Insert consent language here

PART A: Background Information (Note: To be completed before the interview, if possible)

Respondent was the person to complete the survey:

- ☐ Yes
- ☐ No

Respondent's position (from the survey) or if not the person who completed the survey what is the respondent's position within the agency? ______________________________

Respondent's agency: _______________________________________

Respondent is aware of the C.L.E.A.R. Program (If no, describe the C.L.E.A.R. Program):

- ☐ Yes
- ☐ No

PART B: Interview Questions

Section I: Awareness and Attitudes

We will now ask some questions relating to overall awareness of the problem of opioid overdose in the communities you serve, knowledge of the C.L.E.A.R. Program, and your agency's ability to best serve your community.

[CFIR construct: Knowledge & Beliefs (Characteristics of Individuals)]

1. What is your understanding of opioid overdose in the communities your agency serves? What is your understanding of opioid overdose in the community?

2. How familiar are you with the C.L.E.A.R. Program? What is your perception of the impact it can have on your work?

[CFIR construct: Access to Knowledge & Information]

3. If you have questions about the C.L.E.A.R. Program who would you ask or how would you seek out that information?

[CFIR construct: Tension for Change and Implementation Climate (Inner Setting)]

4. Based on your experience, what do you see as the major unmet needs or gaps in the public health infrastructure to address opioid overdoses in the communities your agency serves? What has your agency done in the past year to address those gaps?

5. Do you think there is a strong need for collaborations to address opioid overdose? Why or why not? Do others in your agency share this view?

Section II: Collaboration

In this section we will ask questions about your agency's history with and interest in collaborating with programs that address opioid overdose, such as the C.L.E.A.R. Program, and the benefits and barriers to those collaborations.

[CFIR construct: Complexity (Intervention Characteristics) (Structural Changes, Inner Setting)]

6. How well does the C.L.E.A.R. Program fit with existing work processes and practices in your agency and/or community? What kind of systems level changes will need to happen in order to accommodate collaboration with the C.L.E.A.R. Program?

7. How would collaboration with the C.L.E.A.R. Program impact your work or the work of your agency? To what extent will collaboration help you achieve certain outcomes related to opioid overdose?

[CFIR construct: Relative Advantage/Competition (Intervention Characteristics)]

8. If you could implement any intervention in the communities served by your agency to address unmet needs of opioid overdose, what would it be?

9. Does the C.L.E.A.R. Program present any challenges and/or advantages that will allow you to address the unmet needs you've identified? Are there other existing programs or collaborations that would fill these unmet needs other than the C.L.E.A.R. Program?

[CFIR construct: External Policies & Incentives (Outer Setting)]

10. How will the C.L.E.A.R. Program impact your agency's ability to meet strategic goals related to opioid overdose?

11. How will the C.L.E.A.R. Program impact your agency's ability to meet local, state, or national measures, policies, regulations, or guidelines around opioid overdose?

[CFIR construct: Peer Pressure (Outer Setting)]

12. To your knowledge are other agencies collaborating with the C.L.E.A.R. Program? If so, can you tell me anything about how that experience has been?

13. To your knowledge are other agencies collaborating with other agencies to address opioid overdose in communities served by your agency? If so, what other agencies are serving as collaborators and how has that experience been?

Section III: Engagement

In this section we will ask you about factors that foster engagement with opioid overdose programs, such as the C.L.E.A.R. Program, and how your agency would be best served by engagement with these programs.

[CFIR construct: Engaging (Process)]

14. What were/would be the key factors that prompted/would prompt your involvement with the C.L.E.A.R. Program?

15. Who were/would be the key individuals who have to get on board with collaborating with the C.L.E.A.R. Program? How easy/difficult will it be to engage those key individuals?

[CFIR construct: Client Needs & Resources (Outer Setting)]

16. How do you think the communities served by your agency will respond to you collaborating with the C.L.E.A.R. Program? Will they realize the collaboration?

[CFIR construct: Implementation (Inner Setting)]

17. How receptive are people within your agency to working with the C.L.E.A.R. Program? To working with other programs focused on opioid overdose?

Part IV: Communication

In this section we ask about successful outcomes of collaboration, how you want those outcomes communicated, and the supports you would need for sustainability around addressing opioid overdose in your community.

[CFIR construct: Evidence Strength]

18. When addressing opioid overdose in the community served by your agency, what outcomes matter most to you? How would you judge if the program is successful based on those outcomes?

[CFIR construct: Reflection (Process)]

19. What kind of information have you collected about opioid overdose programs in the communities served by your agency? How have you used this information?

[CFIR construct: Sustainability]

20. To what extent (or in what capacity) would you want the C.L.E.A.R. Program to be sustained to address opioid overdose? Are there other programs with similar missions that you would want to be sustained?

21. What resources would you need to ensure sustainability of programming around opioid overdose? Does the C.L.E.A.R. Program offer those resources? Why or why not?

Conclusion

Thank you for your time. We have completed our questions. Please let us know if you have any questions or additional information you would like us to consider that we didn't discuss today.
